# Some Diseases of the Male Genital System

**Published:** 1907-12-21

**Authors:** 


### SOME DISEASES OF THE MALE GENITAL SYSTEM.

### I.—HYDROCELE OF THE TUNICA VAGINALIS.

By the term vaginal hydrocele we mean a collection of fluid in the tunica vaginalis itself or in a sac connected with it. Keeping strictly to the terms of this definition we can recognise four varieties of vaginal hydrocele: (1) vaginal hydrocele proper, in which the tunica vaginalis alone is distended with fluid; (2) congenital hydrocele, in which the tunica vaginalis contains fluid, but the cavity opens at its upper extremity into that of the peritoneum; (3) infantile hydrocele, in which not only the tunica vaginalis, but the funicular process also, is distended with fluid, and these are shut off from the peritoneum by a constriction at some point on the cord, generally at the external abdominal ring; and (4) hydrocele in the tunica of a retained testis.

The method of causation of the ordinary vaginal hydrocele is even now a matter of no little doubt. There can be no question that an appreciable proportion of these cases is secondary to disease of the testis itself, such as tuberculous, malignant or syphilitic disease. This class of case Mr. Jacobson, in his text-book on "Diseases of the Male Organs of Generation" calls "Secondary Symptomatic Hydroceles." But there is another and much larger class, in which a hydrocele is present without any discoverable concomitant disease of the testes or cord. These we are in the habit of calling "Idiopathic Hydroceles." It is manifest that the use of the word idiopathic is in itself a confession of ignorance. It is nothing more or less than an ingenious way of saying that we do not know or understand. It is easy to say there can be no such thing as an idiopathic hydrocele, any more than there can be an idiopathic tetanus. Before our knowledge of bacteriology was as complete as it is to-day (and even now it is probably only in its infancy) we believed that there were cases of tetanus of idiopathic origin, that is to say there were cases whose causation could not be explained on any known grounds. To-day we realise that tetanus is invariably the result of a definite infection. Can we say, therefore, that there is such a thing as an idiopathic hydrocele? Let us take for a moment the analogy of effusions into the pleural membrane. Until quite recently it has been thought (and by some it is still thought) that there are cases of pleural effusion which make their appearance suddenly and without apparent cause in young adults—a so-called idiopathic pleurisy. But by watching a series of such cases we have come to recognise that the patients who suffer from such a condition develop sooner or later, in some cases several years later, the definite symptoms of tuberculous disease. So constant is the association of the two conditions that, although bacteriological proof of tuberculosis can rarely be obtained from the pleural effusion, it is fair to argue that the pleural effusion was in itself an early manifestation of tuberculous infection. If, then, we say there is no such thing as an idiopathic hydrocele of the tunica vaginalis, are we prepared to put forward a reasonable alternative hypothesis to meet the facts of the case?

It has been suggested that all vaginal hydroceles are really the result of a recent or remote inflammatory process, either bacterial or traumatic. Such a cause can be definitely established in cases of acute hydrocele which come on directly after a blow or some acute infection. But it is not so easy to trace the connection in the more common chronic variety. It has been said that if the wall of a chronic hydrocele be examined microscopically there can always be found some evidence of inflammatory change. This is undoubtedly the case where the condition is secondary to disease of the testis; and it is demonstrable in cases where the scrotum has been subjected to constant irritation or pressure, as by an ill-fitting truss. But it does not advance our knowledge much to say that all cases are inflammatory in origin if the original cause of the inflammation cannot be found. Some authorities have fallen back on the idea that idiopathic hydrocele is always syphilitic in origin, and is secondary to changes produced by this disease in the wall of the tunica vaginalis, apart from disease of the testis itself, while others suggest that the trouble is due to urethral infection, and that inflammation of the tunica vaginalis is the terminal phase of the backward spread of the infection. In favour of the inflammatory origin of vaginal hydrocele are the facts (1) that the fluid contains a greater amount of albumen than is generally found in the fluid of a passive effusion, and (2) that hydrocele of the tunica vaginalis is not a common symptom in diseases associated with passive effusions in other parts—namely, nephritis and advanced cardiac disease.

Mr. Jacobson inclines, however, to the view that vaginal hydroceles start as a passive effusion, and that they may be subjected to irritation later when there is a definite collection of fluid, and thus appearances of inflammatory change in the wall of the tunica may be found under the microscope. The insidious onset of the condition is, he thinks, in opposition to any inflammatory origin which would be associated with an acute onset, and, further, his personal investigation did not tend to prove the existence of old or recent inflammation in this condition.

